# The Role of Methanogenic Archaea in Inflammatory Bowel Disease—A Review

**DOI:** 10.3390/jpm14020196

**Published:** 2024-02-10

**Authors:** Agata Anna Cisek, Edyta Szymańska, Tamara Aleksandrzak-Piekarczyk, Bożena Cukrowska

**Affiliations:** 1 Department of Pathomorphology, The Children’s Memorial Health Institute, Av. Dzieci Polskich 20, 04-730 Warsaw, Poland; 2 Department of Gastroenterology, Hepatology, Nutritional Disorders and Pediatrics, The Children’s Memorial Health Institute, Av. Dzieci Polskich 20, 04-730 Warsaw, Poland; edyta.szymanska@ipczd.pl; 3 Institute of Biochemistry and Biophysics, Polish Academy of Sciences, Pawińskiego 5a, 02-106 Warsaw, Poland; tamara@ibb.waw.pl

**Keywords:** methanogenic archaea, methanogens, IBD, inflammatory bowel disease, Crohn’s disease, ulcerative colitis, pediatric diseases

## Abstract

Methanogenic archaea are a part of the commensal gut microbiota responsible for hydrogen sink and the efficient production of short-chain fatty acids. Dysbiosis of methanogens is suspected to play a role in pathogenesis of variety of diseases, including inflammatory bowel disease (IBD). Unlike bacteria, the diversity of archaea seems to be higher in IBD patients compared to healthy subjects, whereas the prevalence and abundance of gut methanogens declines in IBD, especially in ulcerative colitis. To date, studies focusing on methanogens in pediatric IBD are very limited; nevertheless, the preliminary results provide some evidence that methanogens may be influenced by the chronic inflammatory process in IBD. In this review, we demonstrated the development and diversity of the methanogenic community in IBD, both in adults and children.

## 1. Introduction

Inflammatory bowel disease (IBD) is a term that describes disorders involving chronic inflammation of gastrointestinal tissues. Its importance has been increasing in the recent years, both due to its higher prevalence worldwide [1] and higher treatment costs. It is estimated that over 0.3% of the human population of developed counties suffers from IBD, and there is an upward trend in the incidence rates in various regions of the world [2]. This is related to economic development, dietary changes, and improving economic status [3]. Moreover, the diagnostic possibilities are much better in developed countries than in developing countries, which may also result in better diagnosis of IBD. In the United States alone, the number of people affected by this disease is over 1.6 million, and the annual medical and sick leave expenses is reaching more than $1 billion [4]. The incidence in Europe and North America is estimated at 40–50 and 3.1–14.6 cases per 100,000 inhabitants per year, respectively [5,6].

IBD includes ulcerative colitis (UC) and Crohn’s disease (CD). CD is characterized by an extensive inflammation throughout the entire intestinal cross-section and is usually located in a distal part of the small intestine [7], but may also affect the colon or small intestine and colon together [4]. UC mainly affects the rectal mucosa, where it usually begins [7], and may spread proximally into the colon [8].

The etiopathogenesis of IBD is not fully understood [9], although several links to this disease are well-documented. These include a combination of factors: human genetics, immunology, the environment, and microbiology [10,11]. Commensal microorganisms can be a source of antigens triggering IBD [12], but there are reports directly linking some evidently detrimental bacteria with development of IBD, of which Fusobacteria and *Enterobacteriaceae*, particularly adherent-invasive *Escherichia coli* (AIEC), seem to be among the most important taxa [13,14,15]. Increased amounts of *Candida* spp. and *Malassezia* spp. have also been reported in IBD patients [16,17,18], as have some viruses from the Caudovirales, *Hepadnaviridae*, and *Herpesviridae* clades, such as cytomegalovirus, whose presence contributes to some life-threatening complications [19].

On the other hand, there are bacteria such as *Lachnospiraceae*, *Roseburia*, *Eubacterium rectale*, *Ruminococcus*, *Clostridium*, *Faecalibacterium* and other butyrate producers that may play a protective role, and whose abundance is usually decreased in IBD [20]. However, it should be noted that the role of these bacteria may be strain-specific [21]. In general, the loss of microorganisms producing butyrate (a signaling molecule in the mitochondrial gene expression) and subsequent reduction in this compound in the gut triggers a cascade of adverse events, including deregulation of host mitochondrial activity and an increased production of reactive oxygen species, which eventually allows for translocation of microorganisms and toxins across the epithelial barrier [20]. Moreover, the oxidative stress in the gut prompts a functional adaptation of microorganisms, while leading to microbial dysbiosis, which in turn triggers mucosal inflammation [21]. In conclusion, the abnormal host–microbiota crosstalk and microbial dysbiosis have been demonstrated to play a key role in the pathogenesis of IBD [20,21].

The role of bacteria, fungi, helminths, and viruses in the development of IBD has been extensively reviewed elsewhere [22]. Here, we focus on a forgotten and underestimated member of the gut microbiota, i.e., the methanogenic archaea. Moreover, since children are believed to be the best model for studying the pathogenesis of IBD [20], our work aims to provide as much detail as possible on this age group.

## 2. Methanogenic Archaea in Health and Disease

Archaea were originally known as inhabitants of extreme environments of high salinity, high temperature, and acidity [23], such as thermal springs and deep-sea hydrothermal vents [24]; only later, around 1968, were single species isolated from the human gastrointestinal tract [25]. A few decades later, archaea became known as a part of the physiological intestinal microbiota [26]. Archaea have also been reported in other compartments of human body, such as the oral cavity, nose, respiratory tract, vagina, and skin [27,28]. Currently, archaea are reported in 42 to 100% of fecal samples of the human population, depending on the individuals examined [29,30,31]. Archaea are estimated to account for between 0.1 and 21.3% of the total gut microbiota and are collectively known as the gut archaeome [9,31]. High-throughput sequencing analyses of intestinal samples indicated the presence of the following methanogenic orders: Methanobacteriales, Methanomassiliicoccales, Methanomicrobiales, Methanosarcinales, Methanococcales, and Methanopyrales [26,32]. The non-methanogenic archaeal taxa have also been reported in far fewer cases, and included *Caldisphaera* (order Acidilobales), *Thermogymnomonas* (order Thermoplasmatales), and the orders Archaeoglobales, Desulfurococcales, Natrialbales, Nitrososphaerales, Sulfolobales, Thermococcales, Thermoproteales, Haloferacales, and Halobacteriales [19,26,32,33]. Interestingly, some of these include halophilic archaea, whose abundance in Koreans has been related to the consumption of fermented seafood [31,34,35].

Typically, ca. 90–99% of detected gut archaea are methanogens [27,36]. The methanogenic archaea constitute about 10% of the total anaerobic community [37]. There are three key methanogenic taxa in the human intestine: *Methanobrevibacter smithii* (*Mb. smithii*), *Methanosphaera stadtmanae* (*Ms. stadtmanae*), and Methanomassiliicoccales [32,38]. The prevalence of methanogens increases with the age of humans, with the diversity of methanogens believed to be most pronounced in extreme age groups, i.e., children and the elderly [29,33,39].

For a long time, it was assumed that methanogens begin in children not younger than 2–3 years old [40], but we now know that actual colonization begins much earlier, immediately after birth or even in the fetal period [41,42]. A study in India reported a substantial colonization of neonates in post-weaning children by *Methanobrevibacter* spp. with a frequency of up to 98% for newborns (of which 88.7% were positive specifically for *Mb. smithii*) and 96% for young children aged 6 months to 2 years [33]. Moreover, methanogens (mostly *Mb. smithii*) were reported in the gastric juice of all one-day-old newborns (n = 50) from France [41] and in 90.9% of meconium samples collected from 33 preterm neonates before the first feeding, most of whom were born by cesarean section from mothers who had not been exposed to any antibiotics during pregnancy [43]. The specificity of delivery and lack of feeding prior to sampling challenges the common belief that colonization by methanogens occurs during vaginal birth or the breastfeeding period [41,44]. These new findings may suggest an in utero colonization, possibly through the placenta, amniotic fluid, or blood [43]. Interestingly, one study involving children from birth to one year of age demonstrated that methanogens were detected in the first days of life and disappeared by the second month of life [45]. This transient occurrence of methanogens may be related to the fact that the gut microbial diversity of young infants reduces as a result of breastfeeding [46]. Data are lacking on taxa other than *Mb. smithii* colonizing the intestines of neonates.

In older age groups, in addition to *Mb. smithii* (which was detected in 78 to 88% of school-aged children) other taxa start to emerge. *Ms. stadtmanaea* was found in 8 to 11% and *Methanomassiliicoccus luminyensis* (*Mm. luminyensis*) in 1% of European school-aged children [47,48,49]. More recent data from our research group report slightly different percentages in children aged from 4 to 10 years. In these cases, the prevalence of *Mb. smithii*, *Ms. stadtmanae* and Methanomassiliicoccales were 80%, 47%, and 13%, respectively [50]. The order Methanomassiliicoccales is a broad taxon that includes *Mm. luminyensis* among others, hence the differences between reported numbers [51]. Interestingly, both children and adults demonstrate a marked increase in the prevalence of Methanomassiliicoccales with age [47,50,52]. Moreover, in individual cases Methanomassiliicoccales may dominate quantitatively over Methanobacteriales (including *Mb. smithii*) [50,51].

In adults, *Mb. smithii* was found in 64%, 75–89%, 96%, and even in 100% of the population depending on the individuals studied [26,52]. Data on the prevalence of *Ms. stadtmanae* are also conflicting, as this methanogenic species is estimated to be present in 30 to 90% of adults [30,53]. Notably, the study reporting the highest prevalence of *Mb. smithii* also showed that only 17% of adults and 25% of elderly people aged 70 to 90 tested positive for *Ms. stadtmanae* [52]. Another important methanogen species, *Mm. luminyensis*, was shown to inhabit 4% of adults [47], whereas at the genus level, the prevalence of *Methanomassiliicoccus* ranged from 1 to 25.7% [54]. Interestingly, a small study involving only 10 individuals between the ages of 25–50 showed a 100% prevalence of Methanomassiliicoccales [30], an order that comprises two key genera, i.e., the free-living *Methanomassiliicoccus* and the host-associated “Candidatus *Methanomethylophilus*”. The latter were found to occur in humans even more frequently than *Methanomassiliicoccus*, with a prevalence ranging from 0.5 to 41.7% [54]. In the elderly, the prevalence of Methanomassiliicoccales has reached 40% [52], and despite obvious differences in publications, the general trend is that the prevalence of Methanomassiliicoccales increases with age [47,52], which has not been observed in other methanogenic taxa.

As for absolute values, the abundance of total methanogens, their numbers are also increasing with age. Children at the age of 30 months have 10^3^–10^4^ methanogens per gram of fecal dry matter, whereas in 5-year-olds, this value increases to 10^4^–10^8^ cells per gram [29,50]. In adults, the methanogens can account over 10^10^ cells per gram [55].

The presence of methanogens is strongly related to measured levels of methane in the breath. It is estimated that 15% of Japanese citizens and up to 70% of rural Africans exhale methane, whereas in the Western countries ca. 40–60% of adults are methane producers [56]. The detection rate of methane is related to diet, lifestyle [56], and age [57]. Children as young as 3 years old do not produce methane. About 6% of children aged 3 to 4 produce methane, as do 13 to 18% of children aged 7 to 14, 39 to 46% of teenagers aged 14 to 18, and 49% of adults [50,57]. This correlation is not surprising, given that an individual must be colonized by at least 10^8^ methanogen cells per gram of fecal dry weight for methane in breath to be detectable [58]. Non-methane-producing adults are typically colonized by 10^2^ to 10^6^ methanogen cells per gram of feces [59].

The presence of archaea has been shown to exert a bilateral effect on human health—either positive or negative. On the one hand, methanogens participate in the circulation of matter and energy inside the intestine. By lowering the partial pressure of hydrogen, they assure the continuity of intestinal fermentation, indirectly contributing to the production of specific fermentation products such as short-chain fatty acids or vitamins by the intestinal bacteria [60]. Furthermore, there is growing evidence that the archaeal strain *Mm. luminyensis* B10 can be used as a probiotic, as it has the potential to treat metabolic disorders such as atherosclerosis and trimethylaminuria (TMAU; fishy odor syndrome) [61]. This archaeon naturally depletes trimethylamine with hydrogen in the process of methanogenesis [61]. Archaea have been shown to alleviate yet another unpleasant condition: they can reduce odor by oxidizing ammonia secreted through the skin [36,62]. Moreover, celiac disease studies have shown that Euryarchaeota may play a positive role and act as an anti-inflammatory factor in the healthy guts of children [63]. In pediatric patients aged 6 to 10 years, the presence of *Ms. stadtmanae* was also associated with a lower likelihood of asthma, indicating the tolerogenicity of this species in young patients, a slightly lower (though not statistically proven) risk of eczema, sensitization to aeroallergens and food allergens [49]. On the other hand, the presence of certain methanogenic archaea has been linked to periodontitis, brain abscess, vaginosis, diverticulosis, multiple sclerosis, obesity, colorectal cancer, irritable bowel syndrome, and IBD [26,28,64], but whether the presence of methanogens was a cause or consequence of these conditions needs further investigation. Importantly, not a single study has suggested the existence of a clearly pathogenic archaeal strain in humans [36]. So far, only one study describes them as emerging opportunistic pathogens [65].

## 3. The Occurrence of Methanogenic Archaea in IBD

The first reports indirectly linking methanogens to the incidence of IBD date back to the 1980s [57,66]. At that time, it was observed that adults suffering from IBD rarely excrete methane [57,66,67,68,69]. More than 20 years later, with the development of molecular biology techniques [70,71], this link was confirmed more directly, as Scanlan et al. demonstrated a reduced prevalence of total methanogens in adults suffering from IBD. In their study, the prevalence of methanogens (mostly *Mb. smithii*) in UC and CD was 24% and 30%, respectively, compared to 48% of healthy individuals [59]. A more recent study by our research group performed on children aged 3 to 18 years showed a similar tendency [50]. Of the two IBD entities, the lowest prevalence of methanogens was determined in UC, where 83% of children were colonized by total methanogenic archaea, ca. 52% by *Mb. smithii*, 15% by *Ms. Stadtmanae*, and 15% by Methanomassiliicoccales, compared to 100%, 74%, 37%, and 30%, respectively, as reported in control non-IBD children [50]. In contrast, 89% of CD patients were colonized by total methanogenic archaea, about 69% tested positive for *Mb. smithii*, 27% for *Ms. Stadtmanae*, and 13% for Methanomassiliicoccales. Therefore, the incidence of methanogens recorded in the CD and control groups did not differ significantly, which is consistent with the studies of Krawczyk et al. and Chehoud et al. [18,72].

**Figure 1:**
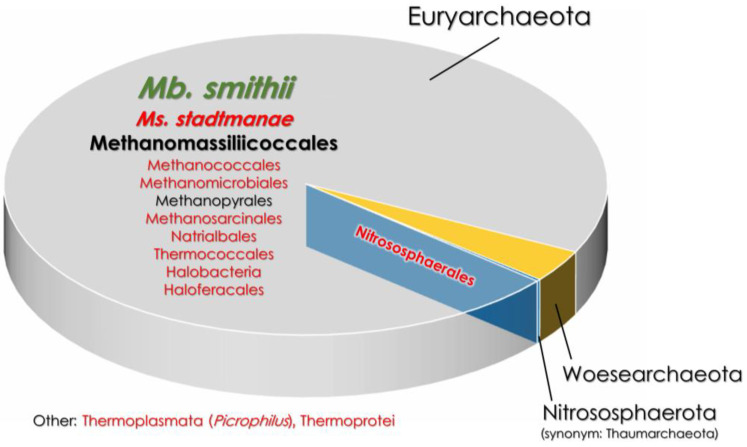
The comparison of gut archaeome in healthy and IBD-affected patients [9,27,73]. The font size indicates the percentage of a given taxon in the gut archaeome. The font color represents taxa that are either increased (red font) or decreased (green font) in IBD patients compared to heathy subjects. *Mb.*—*Methanobrevibacter*; *Ms.*—*Methanosphaera*.

Apart from the lower prevalence of methanogens, there is also a clear link between IBD and a higher diversity of intestinal archaea. The feces of healthy individuals usually have a homogenous composition of archaea comprising a single predominant taxon (Figure 1), such as *Methanobrevibacter* sp. [73]. In contrast, pediatric patients with chronic CD are characterized by an increased archaeal diversity, as non-methanogenic archaea such as Halobacteria have been shown to achieve considerable numbers [73]. Similar results have been observed in adult patients with IBD [74]. Adult CD is also characterized by an increased abundance of Nitrosophaerales and Thermococcales detected in the ileal aspirate [19]. In contrast, in adult UC, most of the detected archaeal lineages of the ileum belonged to Methanococcales, Methanobacteriales, Methanomicrobiales, and Methanosarcinales, therefore UC seemed to be more “methanogenic” than CD [9,19]. In the case of UC, a difference in the archaeal composition between different parts of the intestines was also noted, as the colon was more enriched by the Methanomicrobiales order than the ileum. In CD, no such phenomenon has ever been observed [19].

The discoveries made over the last decade have established that not all archaeal species affect human health in the same way. For example, *Mb. smithii* is considered a commensal species [75], whereas *Ms. stadtmanae* is accused of exerting a rather detrimental effect on its host [12]. *Mb. smithii* accounts for 11.5% of the total intestinal microbiota [12] and constitutes from 94 to 100% of the gut methanogenic population [39,59,76]. *Mb. smithii* tends to be almost as prevalent in adults with IBD as in healthy adults, but in terms of its abundance, these two patient groups differ significantly [12,77]. In healthy adults, the amount of *Mb. smithii* ranged from 10^5^ to 10^9^ cells per gram of dried stool, and only 10^4^ to 10^8^ cells per gram in the IBD patients [12]. Another study reported a decreased prevalence of *Mb. smithii* in the adult IBD patients [59]. In children with CD, the prevalence and abundance values of *Mb. smithii* were not statistically different compared to the control groups [18].

On the other hand, *Ms. stadtmanae* was three times more frequent and numerous in adult IBD patients compared to healthy controls. It was shown that the quantity of *Ms. stadtmanae* in IBD patients was as high as 10^7^ cells per gram of dried stool, compared to a maximum of 10^4^ in healthy adults [12]. Interestingly, a recent study on children with CD reported quite a different trend. There, the prevalence and concentration of *Ms. standtmanae* DNA in stool samples were similar between children with active CD and healthy children, whereas the percentage of carriers and the amount of DNA detected were lowest in CD patients in remission [18]. It should be noted that the latter correlations were not statistically significant.

Methanomassiliicoccales, the last key methanogenic group, is greatly understudied in terms of its role in IBD. As far as we know, its representative—*Mm. luminyensis*—was first described in 2014 [78] and, since then, the role of *Mm. luminyensis* in relation to IBD has yet to be documented. So far, we know that *Mm. luminyensis* is a commensal microorganism exhibiting only low immunogenicity tested in vitro on peripheral blood mononuclear cells (PBMCs) and monocyte-derived dendritic cells (moDCs) [79]. Moreover, its sensitivity profile to the human-derived antimicrobial peptides suggests some adaptation to the intestinal environment [38,79].

## 4. Methanogens in the Pathogenesis of IBD

**Figure 2:**
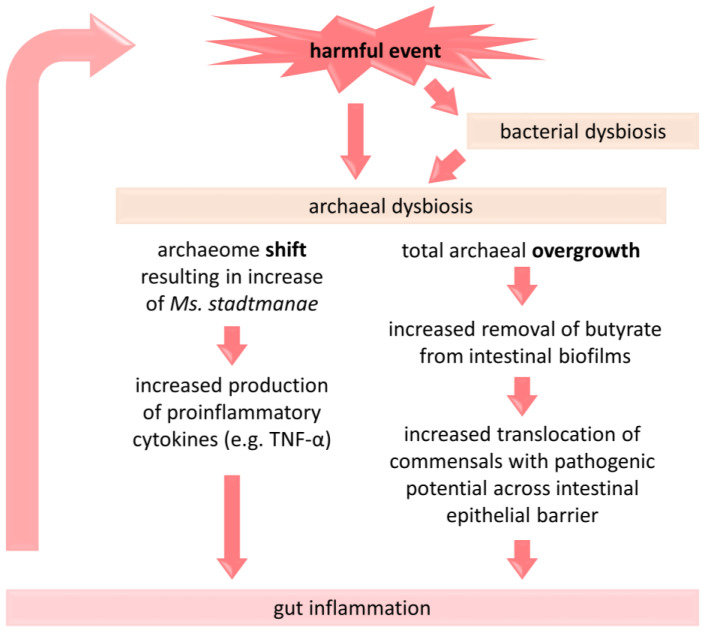
A brief presentation of the proposed link between dysbiosis of methanogenic archaea and the pathogenesis of IBD. A change in the composition of archaea and/or their overgrowth under the influence of detrimental factors (e.g., antibiotic administration, Western-type diet) is associated with an intensification of inflammatory processes in the gut, which may consecutively affect the gut microbiota [9,48,80,81]. TNF-α—tumor necrosis factor alpha.

In the previous chapter we described a higher diversity of archaea occurring in IBD patients compared to healthy subjects, but recent evidence suggests an even higher archaeal diversity the longer the disease lasts. Krawczyk et al. demonstrated that in newly diagnosed pediatric CD, the percentage composition of archaea resembled that observed in healthy children [73]. Over time, both active and inactive forms of long-term CD showed substantial changes in the archaeal composition towards a percentage decrease in predominant Methanobacteria and an increase in other archaea, such as Thermoplasmata, Halobacteria, and Thermoprotei. Similarly, our recent data demonstrated a decrease in the total methanogens in pediatric IBD (particularly in active CD) in older children, i.e., those with longer duration of chronic intestinal inflammation [50]. These two studies may suggest that methanogens were not involved in the induction of IBD, but were influenced by the persistent inflammatory process in the course of IBD [50,73]. It is therefore presumed that prolonged inflammation, chronic diarrhea, and accelerated intestinal transit may (1) contribute to the loss of slow-growing, usually non-motile methanogens [4] and (2) create more favorable conditions for less typical, adventitious groups of archaea [73]. In excess, archaea can deplete large amounts of butyrate from the biofilm lining the intestinal wall. As a result, the intestinal epithelial barrier becomes more permeable, and any commensal microorganisms coexisting in the biofilm can easily enter the intestinal tissues, becoming, as some suggest, endoparasitic [80,81]. As a consequence, inflammation increases, further worsening microbial dysbiosis. Therefore, archaeal dysbiosis and gut inflammation progress in a vicious cycle, especially in the course of IBD (Figure 2). To break this cycle, any measures that reduce inflammation should be of paramount importance. These certainly include maintaining the proper bacterial–archaeal balance and the intestinal production of butyrate, a key regulator of syntrophism between the two groups of organisms [9,20]. As an aside, to date, there has been no clinical trial evaluating the impact of archaeal dysbiosis and IBD.

Apart from the convincing link between IBD and the archaeal dysbiosis, other possibilities of the archaeal involvement in the pathogenesis of IBD have also been the subject of scientific research. Many of these studies were performed on specific species of methanogens. For instance, it was shown that *Mb. smithii* forms biofilms on the surface of intestinal epithelium [82], produces adhesin-like proteins and hyaluronan, all of which generally allows this archaeal species to easily persist in the gut [37]. However, some surface glycans produced by this species have been shown to be immunogenic and induce hyperactive immune responses. This, in turn, may lead in some cases to the development of IBD and other autoimmune-related diseases. This association has been detailed in the case of *Mb. smithii*, whose pseudomurein glycan structures, isolated from a few different strains, revealed the diverse immunogenic potential of these structures tested using monoclonal antibodies [83]. However, as we increasingly understand the interactions between methanogens and bacteria in the gut, it is important to note that there also may be an indirect link between methanogens and the development of IBD. Methanogens produce signaling molecules, e.g., methane or acyl-homoserine lactones. The latter have been shown to be responsible for cell communication [84]. Methanogens have also been proven to promote the growth of fermentative bacteria by decreasing the partial pressure of hydrogen in the gut in the process of hydrogen sink and through symbiotic cross-feeding [85]. It is highly likely that some alteration in the microbiome initiated by methanogens may ultimately affect interactions between the microbiota and the intestinal mucosa or immune cells, as well as the entire inflammatory profile of the gut. For instance, the presence of *Mb. smithi* has been associated with an increased acetogenesis [85,86] and acetate may exert a pro-inflammatory effects [87]. Methanogens may also interact with potentially pathogenic microorganisms, promoting their growth [88]. Moreover, it has been proven that the presence of methanogens and the associated decrease in gut motility (since methane is a signaling molecule) increases mucosal contact time with some toxic metabolic end products, including H_2_S, and therefore promotes their absorption by the intestinal epithelium or induces the intestinal epithelial barrier damage and increased transfer of pathogens [20].

In contrast to *Mb. smithii*, *Ms. stadtmanae* is accused of exerting rather strictly detrimental effect on its host. It strongly induced some inflammatory responses even in the non-IBD healthy individuals [12], whereas in the IBD patients, this species is associated with the persistence of disease [73]. *Ms. stadtmanae* has been shown to be immunogenic to human immune cells such as PBMCs [12] and dendritic cells [36,49], but not to epithelial cells such as Caco-2/BBe, suggesting a pronounced adaptation of this species to the intestinal environment [83]. *Ms. stadtmanae* has been shown to be rapidly phagocytosed by moDC and degraded by endosomal acidification [83]. The release of RNA from *Ms. stadtmanae* (more specifically the ssRNA), acting as a microbe-associated molecular pattern (MAMP), has been shown to induce the secretion of high levels of proinflammatory cytokines (Figure 2) such as interleukin-1 beta (IL-1β), tumor necrosis factor alpha (TNF-α), type-I and type-III interferons via the Toll-like receptors—TLR8 and, to some extent, TLR7. Notably, the secretion of IL-1β depended exclusively on TLR8 [89]. In contrast, DNA from *Ms. stadtmanae* did not yield the same result as its RNA [89].

The RNA-sensing TLRs have been shown to be crucial in various autoimmune and inflammatory diseases, including IBD [90]. In fact, TLR8 mRNA was reported to be upregulated 350-fold and 45-fold in the mucosal inflammatory epithelial cells in UC and CD patients, respectively, compared to controls. However, no differences were observed in TLR8 mRNA levels in lamina propria mononuclear cells between IBD patients and controls [91]. In general, the exposure to *Ms. stadtmanae* contributes to maturation of moDCs and activation of B-cells and T-cells, meaning that both innate and adaptive immune responses are initiated [4]. In contrast, stimulation of PBMCs by *Mb. smithii* has also led to synthesis of TNF-α, but four times lower than stimulation by *Ms. stadtmanae* [12]. It has been hypothesized that both species are specifically recognized by the human immune system, but to different degrees [83]. In summary, individual methanogenic species influence the host’s immunity in different ways. All of the above data should, however, be interpreted with caution as many studies have been conducted exclusively in vitro.

## 5. Conclusions

Methanogenic archaea are part of the physiological intestinal microbiota. The last few years have brought new data on the origins of methanogen colonization in the human intestines and their role in the proper imprinting of the immune system. There is a high probability that methanogens may play a role in non-communicable diseases, but the evidence collected so far regarding the involvement of methanogens in the pathogenesis of IBD is circumstantial. The only certainty is that archaeal dysbiosis occurs in IBD patients, but there is still no convincing evidence on whether methanogens induce pathological mechanisms or are affected by the chronic inflammatory processes that take place in IBD. To date, little is known about the possible role of methanogenic archaea in the initiation and progression of IBD, therefore more research is needed.

## Data Availability

All data are contained within this article.
